# Continuing medical education for attending physicians in anesthesia: Feasibility of an innovative blended learning approach

**DOI:** 10.1097/MD.0000000000037947

**Published:** 2024-04-26

**Authors:** Tian Wang, Yang Zhou, Mao Xu, Ying Deng

**Affiliations:** a Department of Anesthesiology, Peking University Third Hospital, Beijing, China.

**Keywords:** blended teaching, BOPPPS, spinal anesthesia, SPOC, ultrasound

## Abstract

Continuing medical education plays a pivotal role in fostering and upholding the standard of excellence in medicine. Both SPOC (small private online course) and BOPPPS (bridge-in, learning objective, pretest, participatory learning, posttest, and summary) methodologies are rooted in the same educational and learning theories, emphasizing active student engagement, interaction, and feedback. Using ultrasound-guided spinal anesthesia as an exemplar, we aimed to investigate the feasibility of blended teaching (combination of BOPPPS and SPOC) for anesthesiology clinicians and explore trainees’ and trainers’ perspectives towards the innovative method. Twenty-seven attending anesthesiologists were randomly divided into experimental group (n = 14, blended teaching method) and control group (n = 13, traditional teaching method). The questionnaire was administered before and a week post-training. Their operative skills (measured by operation time) were assessed. The students’ cognitive evaluation of the blended teaching mode was conducted in the experimental group. The experimental group demonstrated notably higher theoretical scores compared to the control group [(46.42 ± 5.345) vs (41.92 ± 5.219), t = 2.213, *P* < .05]. The operation time in the experimental group was significantly shorter than that in the control group [(84.79 ± 28.450) seconds vs (114.23 ± 35.607) seconds, t = −2.383, *P* < .05]. Most participants preferred blended learning as it was more effective than traditional learning. Suggestions for enhancement included enhanced online interactivity with trainers and the inclusion of case analysis. Integration of blended teaching incorporating BOPPPS and SPOC methodologies holds promise for enhancing the efficiency of skill training among anesthesiologists. Blended learning may become a viable and well-received option among anesthesia clinicians in China.

## 1. Introduction

Information technology rapid evolution has significantly expanded online education in recent years. The COVID-19 pandemic has curtailed the scope of teaching activities, notably reducing in-person, offline sessions. Despite this shift, continuing medical education demands ongoing practical skills training courses. Consequently, there is an urgent need to explore and implement an effective blended teaching strategy.

The bridge-in, learning objective, pretest, participatory learning, posttest, and summary (BOPPPS) teaching strategy was first proposed by Professor Douglas Kerrin at the University of British Columbia in 1978. This theory comprises 6 stages: bridging-in (B), objective (O), pre-assessment (Pre-assessment), participatory learning (P), post-assessment (P) and summary (S), referred to as BOPPPS.^[1]^ The BOPPPS teaching strategy is grounded in constructivist learning theory, providing a comprehensive framework to attain teaching objectives. Central to BOPPPS is its emphasis on student-centered instruction within an innovative and interactive learning setting. BOPPPS has been applied in health services management teaching,^[2]^ lung cancer courses for clinical medical interns^[3]^ and dental materials courses for predoctoral dental students.^[4]^ By thoughtfully designing the teaching process and reinforcing closed-loop teaching units, it proves beneficial in fostering students’ enthusiasm for learning, enhancing their learning efficiency, and nurturing their capacity for innovative thinking.

Proposed in 2013 by Armando Fox and David Patterson of the University of California,^[5]^ The small private online course (SPOC) effectively merges the attributes of massive open online courses with traditional on-campus classroom instruction. This educational approach offers highly precise course content.^[6]^ Empowered by advanced educational and information technologies, SPOC has transcended the conventional constraints of time and space inherent in traditional teaching models. It has facilitated an open educational environment, amalgamating the strengths of online courses with the interactivity of face-to-face teaching.

SPOC and BOPPPS are consistent on the basis of education theory and learning theory, and both focus on students’ participation, interaction and feedback to improve teaching effect. Blended learning, defined as the combination of traditional face-to-face teaching and online asynchronous or synchronous learning, aims to guide students from the surface to a deep and full understanding of the curriculum, which is a promising education strategy.^[7,8]^ Hence, a framework for a blended teaching mode, drawing from the principles of BOPPPS and SPOC, was developed. This design involved a scientific reconstruction of students’ online and offline learning experiences, offering a novel approach for educational advancement. Currently, this blended teaching method has been successfully implemented in teaching histopathology to dental undergraduates,^[9]^ yielding positive educational outcomes. Nonetheless, within the realm of continuing medical education, the utilization of a blended teaching mode that combines BOPPPS and SPOC remains unexplored territory.

Spinal anesthesia is widely used in lower abdominal, pelvic and lower limb surgery. Conventionally, anesthesiologists rely on anatomical landmarks on the body surface and tactile perception gained from clinical experience to guide needle insertion. However, challenges arise in cases of obesity, deformities, or conditions like ankylosing spondylitis, leading to unclear anatomical localization and potential failure of spinal anesthesia. Utilizing ultrasound guidance offers real-time visualization of deep vertebral bodies and intervertebral spaces, effectively mitigating complications, enhancing the success rate, and boosting patient satisfaction.^[10–12]^ Nonetheless, the adoption of ultrasound-guided spinal anesthesia remains limited. Anesthesiologists’ reluctance primarily stems from a lack of experience, apprehension regarding associated risks, and perceived technical complexity. Training courses encounter challenges due to prolonged teaching durations and intricate subject matter, often resulting in reduced attention and subpar learning outcomes. Thus, the imperative need arises to craft a well-structured and practical training curriculum for ultrasound-guided spinal anesthesia.

The objective of this study is to implement a blended teaching approach integrating BOPPPS and SPOC methodologies within the training curriculum for anesthesiologists focusing on ultrasound-guided spinal anesthesia. We hypothesize that employing blended teaching techniques based on BOPPPS and SPOC will notably enhance the teaching quality.

## 2. Methods

### 2.1. Ethics approval and consent to participate

All methods were carried out in accordance with relevant guidelines and regulations. The experimental protocol was approved by the Intuitional Ethical Committee of Peking University Third Hospital. Written informed consent was obtained from all participants in accordance with the Declaration of Helsinki.

### 2.2. Preparation before training

The instructional focus centered on ultrasound-guided spinal anesthesia, with a tailored syllabus encompassing fundamental anatomical understanding, ultrasound images showcasing various spinal sections, and procedural techniques addressing specific conditions like scoliosis. Faculty recorded comprehensive operation videos and ultrasound-guided spinal anesthesia imagery. To gauge students’ baseline knowledge, theoretical proficiency, and prior practical experience, a pre-assessment questionnaire was created using the “Questionnaire Star” website. Additionally, a post-assessment questionnaire was designed to evaluate the efficacy of the teaching approach and students’ cognitive assessment of the blended learning model.

### 2.3. Subjects and methods

Twenty-seven attending physicians from Peking University Third Hospital were randomly assigned to either the experimental group (n = 14), utilizing a blended teaching strategy, or the control group (n = 13), employing traditional teaching methods.

The control group received instruction through PowerPoint presentations, operation videos, spinal teaching equipment, and 3D body anatomy software. Practical skills training involved simulated teaching equipment at the clinical skills training center, enhancing their operational capabilities.

**Figure 1.**
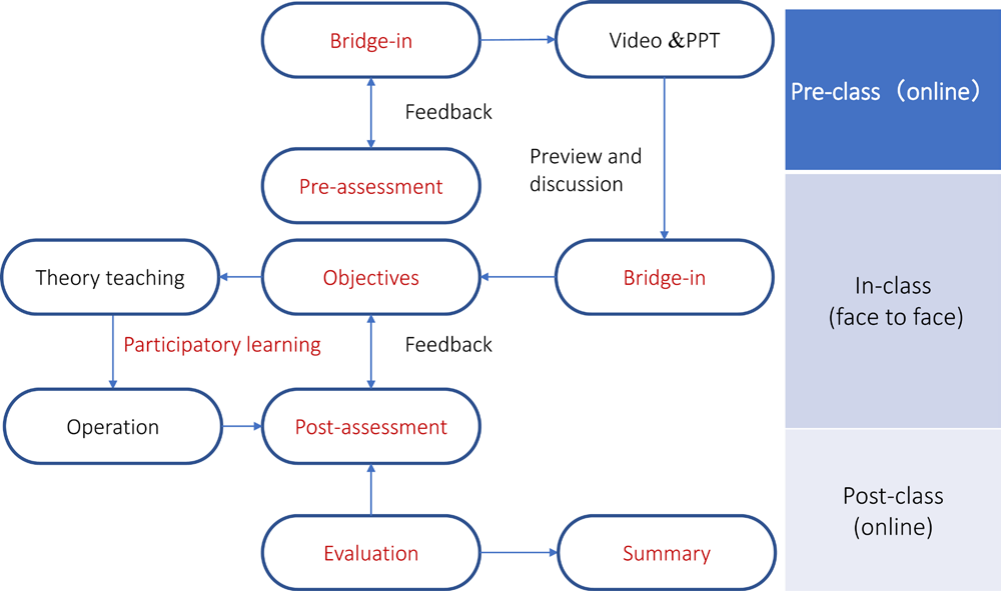
The flowchart of the blended teaching model based on BOPPPS and SPOC. BOPPPS = bridge-in, learning objective, pretest, participatory learning, posttest, and summary, SPOC = small private online course.

The experimental group underwent a blended teaching approach, as illustrated in Figure 1. A private online WeChat group was created to provide online learning support monitored by 2 tutors and for peer discussion. The tutors were senior attending anesthesiologists with expertise in ultrasound-guided spinal anesthesia and had considerable experience in skill training. Firstly, a questionnaire was administered to evaluate theoretical knowledge and operational experience of students (pre-assessment). Subsequently, students received PPT materials and operation videos (the first phase of bridge-in), followed by organized online discussions to address student inquiries (the first phase of participatory learning).

In the offline sessions, clinical scenarios such as scoliosis or severe obesity was showcased and comprehensive clinical status was analyzed to emphasize the significance of ultrasound-guided spinal anesthesia (bridge-in). Students were guided by tutors to focus on learning objectives (probe orientation, basic views, transverse and paramedian sagittal scan, and accurate identification). For participatory learning, theoretical content was presented by tutors who engaged students through watching videos, using 3D body anatomy software, peer interaction and discussions. Practical skills training involved simulated teaching equipment. Peer interaction and discussions were encouraged. Post-training, the online learning group facilitated the sharing of challenging clinical problems and cases among participants.

### 2.4. Evaluation

Operation durations required to locate positions in the simulated teaching equipment were documented for all participating students. To assess the learning outcomes, a theoretical examination was conducted using an online post-assessment questionnaire. Moreover, within the experimental group, students’ cognitive evaluation of the blended teaching mode was assessed via an online survey.

### 2.5. Statistical analysis

All statistical analysis was performed using SPSS, version 27.0 (SPSS, Chicago, IL; SAS, Version 9.3, SAS Institute, Inc., Cary, NC) with the significance level set at a *P*-value not exceeding 0.05. Measurements and endpoints were compared using the *t* test or Mann–Whitney U test as appropriate for continuous variables and the Chi-square test for categorical variables. *P* < .05 was considered statistically significant.

## 3. Results

Table 1 displays the baseline characteristics of students in both groups, revealing no significant differences. In the experimental group, educational analysis revealed that 21.4% held bachelor degrees, 64.3% held master degrees, and 14.3% possessed doctorates. Comparatively, the control group comprised 15.4% bachelor degree holders, 69.2% master degree holders, and 15.4% doctorate holders. Notably, there was no significant distinction in educational distribution between the groups. Regarding comprehension levels, 30.8% of the experimental group and 28.6% of the control group demonstrated a strong grasp of theoretical knowledge pertaining to ultrasound-guided neuraxial anesthesia. Similarly, 14.3% of the experimental group and 15.4% of the control group exhibited a proficient understanding of key operational skills. No statistically significant variance in self-assessed theoretical knowledge or operational skill levels was observed between the 2 groups. Pre-training theoretical scores were recorded as (24.64 ± 9.499) for the blended teaching group and (26.53 ± 11.251) for the traditional teaching group, indicating no significant discrepancy between them (t = −0.474, *P* > .05).

**Table 1:** Characteristics of students and homogeneity tests between the 2 groups.

Variables	Experimental group (n = 14)	Control group (n = 13)
Male, n (%)	5 (35.7)	5 (38.5)
Age, yr	32.6 ± 4.3	33.7 ± 4.5
Degree, n (%) Bachelor Master Doctor	3 (21.4) 9 (64.3) 2 (14.3)	2 (15.4) 9 (69.2) 2 (15.4)
Work experience, yr	6.4 ± 3.7	5.7 ± 4.6
Self-evaluation, n (%) Theoretical Operation skills	4 (28.6) 2 (14.3)	4 (30.8) 2 (15.4)
Theoretical scores		
Pre-assessment	24.64 ± 9.50	26.53 ± 11.25
Post-assessment	46.42 ± 5.35	41.92 ± 5.22
Operation time, sec	84.79 ± 28.45	114.23 ± 35.61

Data are reported as mean ± SD or number (%), where appropriate.

*P < .05.

Following the training, the theoretical score of the experimental group surpassed that of the control group engaged in traditional teaching methods (46.42 ± 5.345 vs 41.92 ± 5.219, t = 2.213; *P* < .05). Regarding the operational aspects, the operation time for the experimental group was notably shorter than the operation time of the control group (84.79 ± 28.450 vs 114.23 ± 35.607, t = −2.383, *P* < .05).

### 3.1. The cognition of teaching model and the evaluation of teaching effect

Within the online WeChat group, an electronic questionnaire was disseminated among students in the experimental group, garnering responses from 13 participants. Table 2 presents the survey questions used. All students diligently completed the preliminary course materials, encompassing courseware and instructional videos shared within the WeChat group. Notably, 28.57% of the students proactively engaged in online forums or video searches, while an equivalent percentage sought relevant books and guidelines, showcasing a heightened level of self-initiated learning. This approach effectively stimulated their learning drive. Regarding satisfaction assessment, participants exhibited high contentment with the blended teaching strategy, as depicted in Table 2. In the open-ended section of the questionnaire, trainees offered valuable suggestions to enhance the teaching model. These recommendations included augmenting interaction time during classes, extending the duration allocated for case analyses, incorporating unsuccessful case studies along with comprehensive analyses, and introducing additional simulated models to facilitate students’ practical application.

**Table 2:** Questionnaire of the blended teaching group.

	Question	Score
1	Have you completed the preview before the course?	/
2	Through what way did you conduct the preview?	/
3	Evaluation of theoretical knowledge explanation	9.8 ± 0.4
4	Evaluation of practice training	9.6 ± 0.8
5	Innovation evaluation of training model	9.9 ± 0.3
6	Evaluation of Teacher guiding ability	9.9 ± 0.3
7	Has this course aroused your learning initiative? 1 for none, 10 for the most	9.8 ± 0.4
8	Are you satisfied with the overall teaching?	9.8 ± 0.6
9	Do you have confidence in future clinical practice? 1 for none, 10 for full of confidence	9.2 ± 1.3

Data are reported as mean ± SD.

## 4. Discussion

The COVID-19 pandemic has caused an unprecedented impact on global public health, significantly affecting the realm of medical education.^[13]^ Online teaching offers significant convenience for the continuation of medical education. This study marks the initial exploration of applying a blended teaching method rooted in BOPPPS and SPOC within the skill training of attending physicians in anesthesiology, presenting a promising concept for ongoing professional development in this field.

Within the realm of continuing education training for clinicians, particularly in clinical operational skills, the blended teaching method could present notable advantages. Firstly, online teaching proves convenient for small-group instruction, circumventing the need to compromise patient privacy. Secondly, during in-person sessions, instructors can offer tailored responses to students’ online pre-assessments, previews, and specific queries, providing personalized operational guidance that fosters student engagement. Thirdly, feedback gleaned from post-assessment questionnaires aids in analyzing students’ comprehension of both theoretical and practical knowledge. This feedback allows for timely adjustments, enhancing the long-term efficacy of teaching methodologies.

The new teaching model imposes heightened demands on educators, necessitating a departure from traditional lecture-based pedagogy. Interactive teaching mandates that instructors possess extensive theoretical knowledge and substantial clinical practice expertise. During the pre-teaching assessment phase, teachers must analyze students’ foundational understanding and discern individual needs. Throughout the teaching process, instructors should adeptly guide students in exploring and resolving problems aligned with their interests, fostering active participation in interactive learning. The blended teaching model, rooted in BOPPPS and SPOC, should embody an open teaching design. Teachers are required to infuse their wealth of teaching experience into daily instructional practices while dynamically adapting teaching designs to align with specific content and students’ foundational knowledge. This adaptive approach ensures greater resonance with students’ psychological inclinations and cognitive processes.

## 5. Conclusion

Integration of blended teaching incorporating BOPPPS and SPOC methodologies holds promise for enhancing the efficiency of skill training among anesthesiologists. This approach also contributes to a comprehensive improvement in students’ mastery of theoretical knowledge and operational skills. Blended learning may become a viable and well-received option among anesthesia clinicians in China.

## Author contributions

**Conceptualization:** Tian Wang, Yang Zhou.

**Data curation:** Yang Zhou.

**Formal analysis:** Mao Xu.

**Investigation:** Tian Wang, Mao Xu.

**Methodology:** Yang Zhou.

**Project administration:** Tian Wang.

**Supervision:** Mao Xu, Ying Deng.

**Writing – original draft:** Tian Wang, Yang Zhou.

**Writing – review & editing:** Mao Xu.
